# Dynamic changes in ORC localization and replication fork progression during tissue differentiation

**DOI:** 10.1186/s12864-018-4992-3

**Published:** 2018-08-22

**Authors:** Brian L. Hua, George W. Bell, Helena Kashevsky, Jessica R. Von Stetina, Terry L. Orr-Weaver

**Affiliations:** 1 Whitehead Institute for Biomedical Research, Cambridge, MA 02142 USA; 2 Department of Biology, Massachusetts Institute of Technology, Cambridge, MA 02142 USA; 3 Present Address: Centers for Disease Control and Prevention, 1600 Clifton Rd, Atlanta, GA 30329 USA

**Keywords:** DNA replication, Transcription, Drosophila, Common fragile sites

## Abstract

**Background:**

Genomic regions repressed for DNA replication, resulting in either delayed replication in S phase or underreplication in polyploid cells, are thought to be controlled by inhibition of replication origin activation. Studies in Drosophila polytene cells, however, raised the possibility that impeding replication fork progression also plays a major role.

**Results:**

We exploited genomic regions underreplicated (URs) with tissue specificity in Drosophila polytene cells to analyze mechanisms of replication repression. By localizing the Origin Recognition Complex (ORC) in the genome of the larval fat body and comparing this to ORC binding in the salivary gland, we found that sites of ORC binding show extensive tissue specificity. In contrast, there are common domains nearly devoid of ORC in the salivary gland and fat body that also have reduced density of ORC binding sites in diploid cells. Strikingly, domains lacking ORC can still be replicated in some polytene tissues, showing absence of ORC and origins is insufficient to repress replication. Analysis of the width and location of the URs with respect to ORC position indicates that whether or not a genomic region lacking ORC is replicated is controlled by whether replication forks formed outside the region are inhibited.

**Conclusions:**

These studies demonstrate that inhibition of replication fork progression can block replication across genomic regions that constitutively lack ORC. Replication fork progression can be inhibited in both tissue-specific and genome region-specific ways. Consequently, when evaluating sources of genome instability it is important to consider altered control of replication forks in response to differentiation.

**Electronic supplementary material:**

The online version of this article (10.1186/s12864-018-4992-3) contains supplementary material, which is available to authorized users.

## Background

Proper control of the DNA replication program is crucial in the maintenance of gene copy number and genome stability. Accordingly, DNA replications programs are tightly coordinated with development, and this coordination is essential for tissue and organism function [1]. However, the mechanisms by which tissue differentiation regulates fundamental aspects of DNA replication including origin specification, origin activation, and replication fork elongation remain unclear.

In metazoans, regions of the genome replicate at distinct times during S phase. Generally, regions containing active genes replicate earlier in S phase than regions with low gene density or repressed genes. The genome is organized into large replication timing domains that correspond to regions of higher-order chromatin structure defined by interaction maps [2]. As embryonic stem cells differentiate, replication timing domains consolidate into larger units, and 20% of the mouse genome changes its time of replication [3]. Although these changes are associated with altered gene expression within a replication timing domain, evidence for causality has not yet been established. Similarly, 20% of the genome differs in replication timing between different Drosophila cell lines [4]. The time in S phase when domains replicate has been proposed to be controlled at the level of replication initiation through differential timing of replication origin activation [4].

Origins have been mapped in metazoans by genome-wide studies that localize replication structures such as short nascent strands or replication bubbles [5]. In Drosophila and human cell lines localization of the binding sites of ORC, a protein complex necessary to load the replicative DNA helicase, has been an alternative approach to identify origins [6–8]. Together these studies have found a higher density of origins in early versus late replication regions and established a link between replication origins and transcription start sites and enhancers [5]. The latter connection appears to be due to a requirement for open chromatin for ORC DNA binding, which may be facilitated by bound transcription factors [7]. In mammalian cells there are multiple origins within each replication-timing domain, producing a zone of potential initiation sites, only some of which are active in a given S phase [9]. Much remains to be understood about the positioning and activation of replication origins.

Far less is known about control of replication forks in eukaryotes. Proteins and signaling pathways that restore stalled forks have been identified in yeast and metazoans [10, 11]. Although late replicating regions could be replicated passively by forks from adjacent origins, it has been proposed that timing of origin firing rather than regulation of fork progression is responsible for differences in replication timing in S phase [2, 7, 12].

Nearly all differentiated tissues in Drosophila increase their DNA content via a modified cell cycle with solely G and S phases, the endocycle. The endocycle can produce polyploid or polytene cells, which differ in that in polytene cells the replicated sister DNA helices are held in register to give a banded pattern in visible chromosomes. Differential DNA replication occurs during the endocycle, resulting in some genomic regions being underreplicated and having reduced gene copy number or others being overreplicated, leading to amplified genes [13]. These differential DNA replication events are developmentally controlled. For example, to date gene amplification has been observed solely in the ovarian follicle cells. In contrast, many tissues contain underreplicated genomic intervals, and these are powerful models to investigate the mechanisms by which origin positioning and firing are controlled. Studies of DNA replication of these underreplicated regions (URs) also provide a unique opportunity to investigate how replication fork progression can be regulated.

Analysis of the Suppressor of Underreplication (SUUR) protein has implicated regulation of replication fork progression as contributing to inhibition of DNA replication at specific genome intervals. The SUUR protein was identified by the requirement for its function for underreplication [8, 14–16]. Notably, this protein has no effect on ORC binding or origin activity [8, 17]. SUUR tracks with and destabilizes replication forks in specific chromosomal regions, properties that can explain its impact on gene copy number [17, 18].

Here we show extensive tissue-specificity of underreplication and investigate the potential contribution of control at the level of replication initiation at ORC sites. Strikingly, although there are constitutive genomic regions that lack ORC, this alone does not account for underreplication. Rather, it appears that active regulation of replication fork progression dictates the extent to which these potential underreplication regions are replicated.

## Results

### Tissue-specific programming of replication

**Fig. 1:**
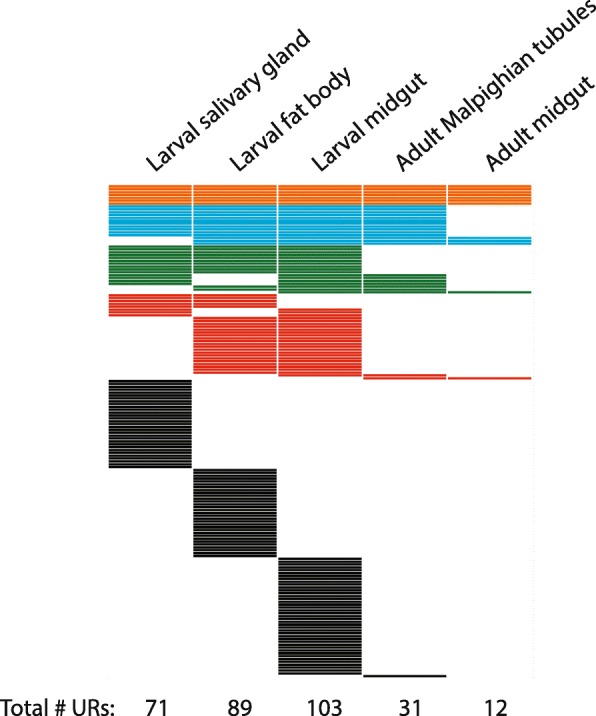
Comparison of UR regions across larval and adult tissues. Each row represents a distinct UR region present in both biological replicates performed for each tissue (172 URs in total across all five tissues). URs shared by all five tissues (orange), four tissues (blue), three tissues (green), two tissues (red), or that are specific to one tissue (black) are shown. A box signifies that region is underreplicated in that tissue

Prior to investigating mechanisms of underreplication we extended our previous analysis of the developmental regulation of differential DNA replication by examining two adult tissues and by changing our statistical method for determining genomic regions with altered gene copy number. Previously our lab used array-based comparative genomic hybridization (aCGH) to profile gene-copy number genome-wide in three polytene tissues isolated from late-3rd instar wandering larvae, revealing a high degree of tissue-specific underreplication [15]. Here we analyzed the Malpighian tubules and the midgut in the adult female (Fig. 1, Additional file 1: Figure S1, Additional file 2: Figure S2). The Malpighian tubules function as the kidney and are an unusual tissue in Drosophila, because of their persistence from the larval through the adult stage where they attain an average ploidy of 168C (Additional file 1: Figure S1). In contrast, the polytene larval midgut tissue is destroyed during pupation and built anew from diploid progenitors to reach ploidy values up to 32C [19–22] (Additional file 1: Figure S1).

Using the previously generated aCGH data sets from the three larval tissues and the two newly profiled adult tissues, we called underreplicated regions (URs) employing a statistical analysis pipeline based on that of Hannibal et al. [23]. Notably, even with this lower stringency statistical UR calling, we found that 96% of the URs in the five tissues analyzed show extensive tissue specificity (Fig. 1, Additional file 3: Table S1). If we calculate the bp that are called as underreplicated 84% (12.2 Mb of 14.5 Mb total) show tissue specificity. Thus, the differentiation state of the tissue impacts the parameters of DNA replication and whether genomic regions are repressed for DNA replication.

### DNA replication and transcription during the endocycle in the larval fat body

We next investigated potential regulatory mechanisms that could explain the extensive tissue specificity of underreplication. Previously, we profiled the genome-wide localization of ORC in the larval salivary gland and found that URs are nearly devoid of ORC binding, indicating that replication initiation largely does not occur in these regions [8]. This would be consistent with differential replication across the genome largely being controlled by initiation of replication, as proposed for replication timing in mammalian cells [2, 12]. To determine if lack of ORC in URs is a general feature, we chose to map the location of ORC binding in a second polytene tissue, the larval fat body. This approach also permitted us to analyze if the difference in URs between these two tissues could be accounted for by differential ORC localization.

**Fig. 2:**
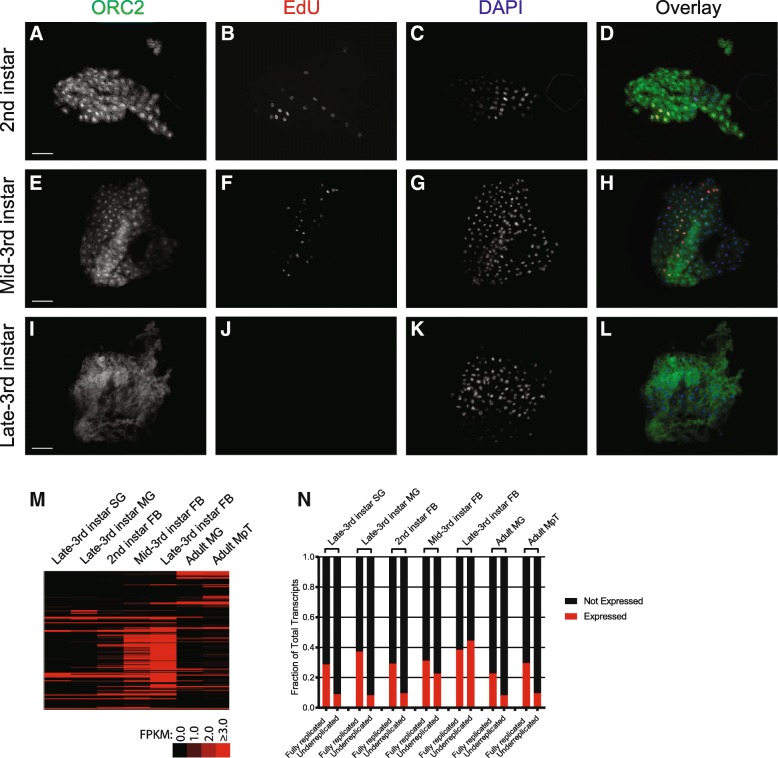
Cytological analysis of replication and dynamics of transcription during larval fat body development. Representative immunofluorescence images of 2nd-instar (**a**-**d**), mid-3rd instar (**e-h**), and late-3rd instar (**i-l**) larval fat body. ORC2 staining (**a, e, i**) is shown as green in the merged images. EdU staining (**b, f, j**) is depicted as red, and DAPI (**c, g, k**) is depicted in blue. Scale bar represents 100 μm. **m,n** Expression of transcripts within UR regions. **m** Expression profile heatmap of the 146 transcripts within the 7 UR regions common to all five tissues profiled by aCGH, including the adult midgut (MG) and adult Malpighian tubules (MpT) (late-3rd-instar larva data from [15]). **n** Fraction of expressed transcripts within the 7 common UR regions and fully replicated regions for the indicated tissues. Only transcripts with FPKM values of ≥3 are considered expressed

Before mapping ORC binding by chromatin immunoprecipitation coupled with high-throughput sequencing (ChIP-seq), we defined the developmental timing of endocycling in the larval fat body. We labeled fat bodies isolated from 2nd-instar, mid-3rd instar, and late-3rd instar larvae with the thymidine analog 5-ethynyl-2′-deoxyuridine (EdU) to detect actively replicating cells and performed ORC2 immunofluorescence studies to visualize the cellular localization of ORC. Whereas all fat body samples isolated from 2nd- and mid-3rd instar larval exhibited EdU-positive nuclei, we failed to detect EdU incorporation in any fat body sample isolated from late-3rd instar larvae (Fig. 2b, f, and j). Additionally, 2nd- and mid-3rd instar larval fat body exhibited strong nuclear localization of ORC2 (Fig. 2a and e). In contrast, ORC2 was not concentrated within the nuclei in late-3rd instar larval fat body (Fig. 2i). These results show that DNA replication ceases by the late 3rd instar stage and that this may be associated with loss of ORC from the chromatin.

Identification of the developmental timing of DNA replication in larval fat body cells revealed that the URs can be repressed for replication but active for transcription. The URs in the adult midgut and Malpighian tubules are repressed for transcription (Fig. 2m, n), as has been reported previously in the larval salivary gland and midgut tissues [15]. Although it was noted that transcription of genes within the URs occurs in the larval fat body, this study was done in late 3rd instar, a developmental stage when we now know that the endocycle has ceased (Fig. 2j). By performing mRNA-seq profiling of 2nd instar and mid-3rd instar fat body we found that transcription is low in the 2nd instar but has initiated by mid-3rd instar (Fig. 2m). Thus in the mid-3rd instar larval fat body both the endocycle and transcription take place, showing that repression of DNA replication across the URs can occur concurrently with transcription. Notably, 33/146 (23%) of transcripts in the URs common to the five tissues are expressed in the mid-3rd instar fat body (Fig. 2m, n). We conclude that, unlike the other tissues examined, the URs in the larval fat body can be repressed for replication but permissive for transcription at the same developmental stages.

### Tissue differentiation regulates genome-wide ORC binding

To analyze ORC binding sites in the fat body genome-wide, we performed ORC2 ChIP-seq on 2nd instar, mid-3rd instar, and late-3rd instar larval fat body in biological replicates and called significant ORC2 binding peaks genome-wide. Consistent with the EdU labeling and immunofluorescent staining of fat body cells, the number and density of ORC2 binding sites detected by ChIP-seq reduced dramatically between 2nd and late 3rd instar larvae (5351 total ORC2 peaks called in 2nd instar, 2212 peaks in mid-3rd instar, and 752 peaks in late-3rd instar larval fat body across two biological replicates). This loss of ORC binding is consistent with the immunofluorescence results showing ORC is cleared from the chromatin and DNA replication is reduced by late-3rd instar (Fig. 2).

**Fig. 3:**
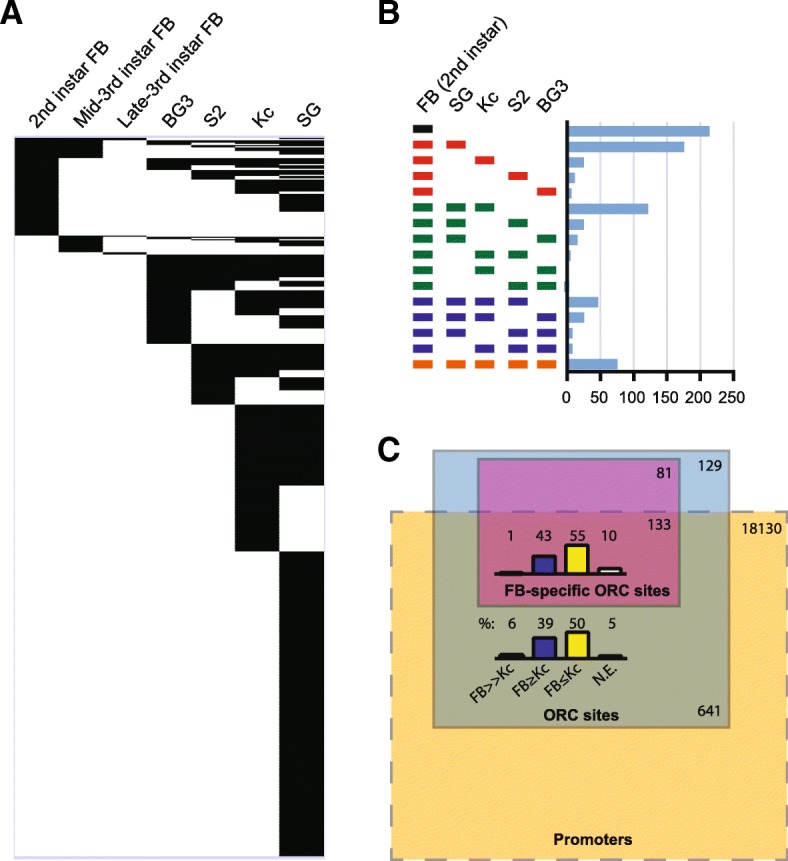
Comparative ORC2 binding analysis across Drosophila tissues and cell culture lines. **a** Comparison of high-confidence ORC2 binding sites (Irreproducible Discovery Rate, IDR < 0.1) from 2nd-instar larval fat body (FB), mid-3rd-instar larval FB, late-3rd-instar larval FB, late-3rd-instar larval salivary gland (SG) [8], Kc cells, S2 cells, and BG3 cells [6]). ORC2 binding sites within 1 kb were treated as overlapping. **b** Comparison of 2nd-instar FB ORC2 sites with late-3rd-instar larval SG, Kc cells, S2 cells, and BG3 cells. 2nd-instar larval FB specific ORC sites (black), 2nd-instar larval FB sites shared with one (red), two (green), three (blue), or all other cell and tissue types (orange) are depicted. **c** Rectangular Venn diagram comparing the relationship between ORC binding and transcription start sites (TSSs) in 2nd-instar larval FB. All transcription start sites within 1 kb of an ORC site were identified, and the percentile rank of the corresponding transcript (in FPKM) was determined from FB and from Kc cell RNA-seq data [46]. The difference in percentile rank (DPR) was then calculated for each transcript between FB and Kc cells. Each transcript was then classified as FB-specific (FB>> Kc, for DPR > 40, black), higher in FB or no difference (FB ≥ Kc, for DPR between 1 and 40, blue), lower in FB or no difference (FB ≤ Kc, for DPR ≤ 0, yellow), or not expressed in FB (N.E., white)

To map the genomic sites of ORC localization in the fat body, we focused on the 2nd-instar larval fat body ORC2 ChIP dataset that had the greatest number of ORC2 peaks. We identified high-confidence ORC2 peaks common to both biological replicates using irreproducible discovery rate (IDR) metric [24] (IDR < 0.1). From this analysis, we obtained 770 distinct ORC2 binding sites. We then compared these sites to those identified in late-3rd instar salivary gland [8] and three Drosophila cell culture lines [6] (Fig. 3a). Of the 770 2nd-instar larval fat body ORC2 peaks, 214 peaks (28%) were unique to the fat body (Fig. 3b). This is consistent with previous findings in the larval salivary gland that each cell and tissue type exhibits a high degree of cell-type specificity [8]. Interestingly, larval fat body and salivary gland shared a markedly higher number of ORC2 peaks (176 peaks, 23%) than any other exclusive pairwise comparison made with the fat body (7–25 peaks) (Fig. 3b). In total, 496 (64%) of the larval fat body ORC2 peaks were also found in the salivary gland (Fig. 3a, b), indicating a high level of overlap in ORC2 binding peaks in these two endocycling tissues isolated from similar developmental time points.

In cultured Kc cells, 64% of ORC binding sites are associated with transcription start sites (TSSs) of actively transcribed genes [25]. In the late-3rd instar salivary gland, 73% of ORC binding sites are within 1 kb of a TSS. However, the expression of the genes associated with ORC binding in the salivary gland is not unique to this tissue, indicating that the tissue specificity of ORC binding is not correlated with active TSSs [8]. This prompted us to examine the ORC localization in the fat body relative to transcription start sites and the activity of their associated genes. 641/770 (83%) of the 2nd-instar larval fat body ORC peaks are within 1 kb of a TSS, with 95% of the genes associated with these TSSs actively expressed in this tissue, consistent with the finding in both Kc cells and salivary gland that ORC binding is enriched at active TSSs (Fig. 3c). 133/214 (62%) of fat-body specific ORC binding sites are within 1 kb of a TSS, but the vast majority of genes controlled by these promoters are not uniquely expressed in the fat body (Fig. 3c). Thus, consistent with results from the salivary gland, tissue-specific ORC binding in the fat body is not correlated with tissue-specific gene expression.

### ORC binding is repressed constitutively across regions that can be underreplicated

**Fig. 4:**
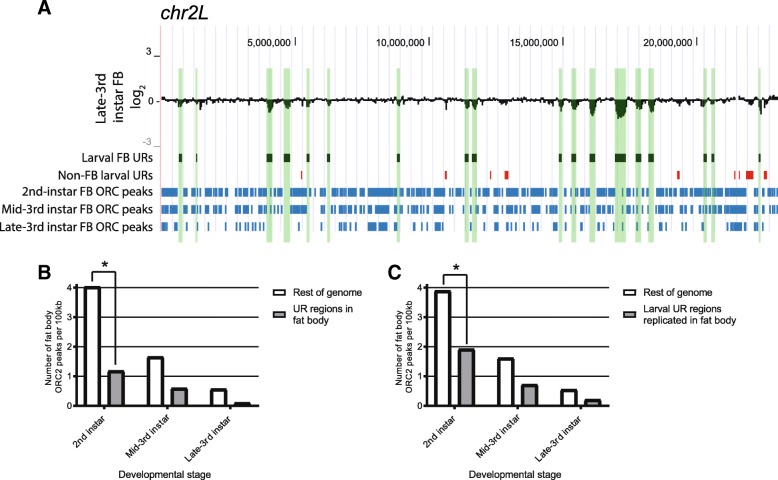
Analysis of ORC binding in the larval fat body (FB). **a** aCGH profile of late-3rd-instar larval fat body (data from [15]) and the peak summit locations of ORC2 in the 2nd-instar, mid-3rd-instar, and late-3rd-instar larval fat body on *chr2L* with dm3 genome coordinates shown. UR regions in the late-3rd-instar larval fat body are denoted as black boxes and are highlighted by green shading. Regions that are underreplicated in other larval tissues but are fully replicated in late-3rd-instar larval fat body are denoted as red boxes. **b** Comparison of the number of fat body ORC2 binding sites per 100 kb within the late-3rd-instar larval fat body UR regions relative to fully replicated regions at different larval stages. * *p* < 10^− 5^. **c** Comparison of the number of fat body ORC2 binding sites per 100 kb within regions that are underreplicated in other larval tissues but are fully replicated in late-3rd-instar larval fat body relative to fully replicated regions at different larval stages. *p < 0.001. For the analyses in panels B and C, to be conservative all ORC2 binding sites were considered, not solely the high confidence set

We next examined ORC distribution in the regions that are underreplicated in the fat body. We found that at all three developmental stages, ORC2 was largely absent in the URs compared to the fully replicated regions of the genome (Fig. 4a, b). To assess the statistical significance of the marked decrease of ORC2 binding in these regions, we randomly sampled each of the 89 fat body URs with the 2nd-instar larval fat body ORC2 peaks dataset and determined the expected number of ORC peaks within these regions by random chance. We found that the low number of observed 2nd-instar ORC2 peaks within the URs was significantly lower than that expected by chance (*p* < 10^− 5^). Thus, similar to the larval salivary gland, ORC binding is largely repressed in URs in the larval fat body. Note that although the density of ORC binding in the URs is significantly lower than in fully replicated regions of the genome (Fig. 4, S4C), not all of the URs are completely devoid of ORC. 40% of URs in the fat body (36/89) and 52% of URs in the salivary gland (37/71) have at least one ORC binding site occupied.

Having mapped ORC binding sites from two polytene tissues, we now could test whether the tissue-specificity of URs could be explained by changes in ORC binding; that is whether regions that were underreplicated in one tissue but replicated in the other would exhibit differential ORC localization. Strikingly, domains that are underreplicated in other larval tissues but replicated in the fat body remain largely devoid of ORC2 binding in 2nd-instar larval fat body (*p* < 0.001) (Fig. 4a, c). We found this to be true also in the salivary gland: regions that were replicated in the salivary gland but underreplicated in other tissues show similarly low levels of ORC2 localization in the salivary gland (Additional file 4: Figure S3, Additional file 5: Figure S4). Moreover, these regions correspond to areas of low density of ORC binding in diploid cells in culture that are known to replicate late in S phase (Additional file 5: Figure S4) [5, 6, 25]. These findings indicate that ORC-repressed domains are constitutive across tissues and mark chromatin domains susceptible to underreplication. Importantly, they exclude the idea that a lack of ORC binding (and hence replication origins) is sufficient to account for tissue-specific underreplication and indicate that other mechanisms must contribute.

For an ORC-free domain, we tested whether its replication in one tissue but underreplication in another tissue could be accounted for by even low-level recruitment of ORC to the region in the tissue in which it is replicated. This is not the case. 34 of the URs in the salivary gland completely lack ORC. 21 of these are fully replicated in the fat body, yet the majority (14) still completely lack ORC in the fat body. Similarly, of the 53 ORC-free URs in the fat body, 36 are fully replicated in the salivary gland, but 25 of these also lack ORC binding in the salivary gland. Thus, the majority of instances of tissue-specific replication of potential UR regions cannot be explained by differences in ORC binding, further supporting the idea that additional mechanisms must contribute to underreplication.

The finding that genomic regions largely devoid of ORC are constitutive across tissue types raised the question of whether these domains lacking ORC correspond to a higher-order organization of the chromatin. One manifestation of this organization is the Topologically Associated Domains (TADs), which have been shown to be conserved between endocycling salivary glands, embryos, and diploid cells in culture, revealing a constitutive organization of the genome [26]. TADs are units of replication timing in mammalian cells [27]. We found that not only are the URs associated with TADs, but they are contained within them (Additional file 6: Fig. S5). Of the 71 salivary gland URs, 83% map completely within a salivary gland TAD, 8% cross a TAD boundary, and 8% are outside of any TAD. Of the total 172 URs in the five tissues, 83% lie within a TAD, 5% cross a TAD boundary, and 12% map outside of known salivary gland TADs. It is intriguing that the vast majority of URs lie inside of TADs, suggesting that the replication domains cannot cross TAD boundaries. The URs that do cross a TAD boundary span two closely spaced TADs, as if the boundary between them did not impact replication and they affect replication as a unit. The URs that map outside of TADs are unusual in being very small, ranging in size from only 2.2 to 17 kb. A correlation between URs and TADs has been noted recently by others as well [28, 29].

### Replication across ORC-repressed regions by control of replication fork progression

**Fig. 5:**
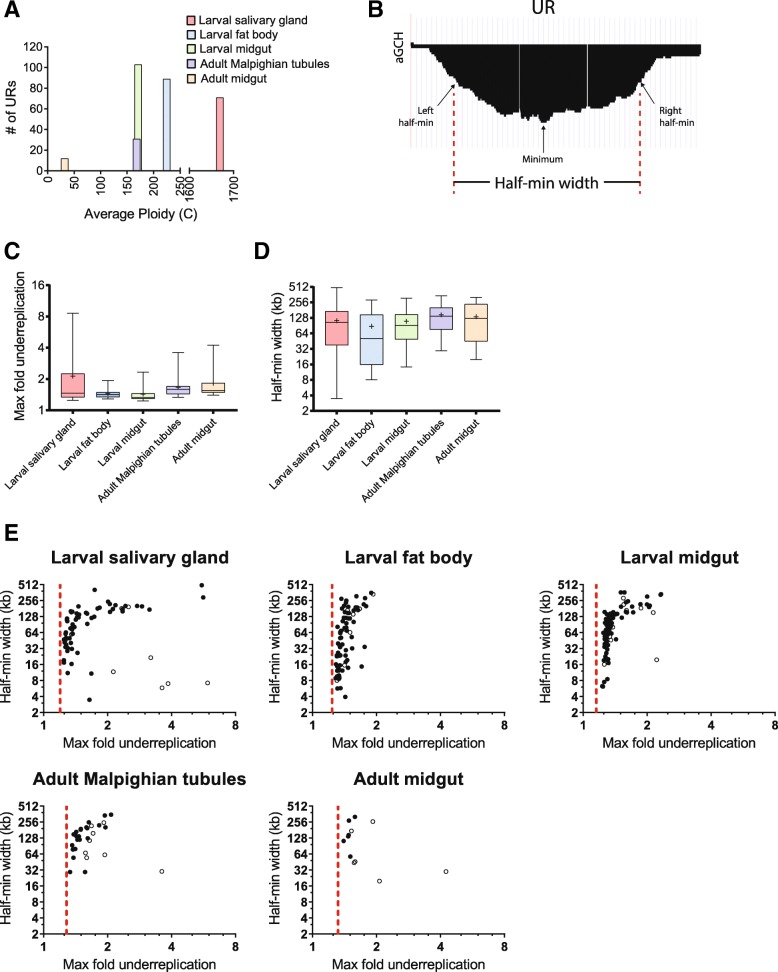
Comparisons between ploidy, UR width, and UR depth. For these analyses the URs identified in both biological replicate experiments were used. **a** Number of URs called in a tissue versus its average ploidy value. **b** For each UR in each tissue, the probe with the minimum log_2_ ratio was determined. The probe to the left of the minimum with half the minimum log_2_ ratio was set as the left half-min position. The probe to the right of the minimum with half the minimum log_2_ ratio was set as the right half-min position. The distance between the left and right half-min positions was determined as the half-min width. The half-min widths or maximum fold underreplication values for a given UR were averaged across biological replicates within a tissue. **c** Box plots of the maximum fold underreplication of the URs identified in each tissue. “+” denotes the mean value of the distribution. **d** Box plots of the half-minimum widths of the URs identified in each tissue. “+” denotes the mean value of the distribution. **e** Maximum fold underreplication versus half-min width for each UR identified in each tissue. The dotted line denotes the fold underreplication cutoff value for calling URs for that tissue. Open circles show URs in pericentromeric regions. Box plot and scatterplots are displayed with log_2_ axes

The observation that the URs are largely devoid of ORC in both the salivary gland and fat body, yet can be replicated in some tissues, presents the paradox of how domains up to 500 kb can be replicated without origins within them. We further analyzed the developmental properties of these URs to provide insights into potential mechanisms by which they could be replicated in some tissues. We investigated how tissue ploidy, and therefore the number of S phases, in the cells relates to the number of URs. If underreplication results from random replication errors then the number of URs would be expected to be higher for tissues that undergo more rounds of DNA replication. In contrast to this prediction, the salivary gland has the highest ploidy of the tissues analyzed, by nearly 10 fold, yet it does not have the highest number of URs (Fig. 5a). Although the fat body, larval midgut and Malpighian tubules have comparable ploidy, they had a range of 89, 103 and 31 total URs, respectively (Fig. 5a). Only the adult midgut showed a correlation with a low number of URs and its low ploidy of 32C.

We quantified the width of the underreplication domain and fold underreplication (minimum copy number) for all of the URs (Fig. 5b). The mean maximum fold underreplication did not correlate with tissue ploidy, except that there were outliers of very low copy number in the salivary gland (Fig. 5c). There was no relationship with the width of the URs and tissue ploidy (Fig. 5d). These results argue against the URs arising from random DNA replication defects, suggesting active developmental control.

The URs are detected as a gradient of decreased copy number. Interestingly, we found that in all five tissues, as expected, wider URs showed a higher level of copy number reduction, although this was most pronounced for the salivary gland (Fig. 5e). In the salivary gland and Malpighian tubules there are several URs that are narrow (half-min widths of 4-32 kb) but have low copy number (open dots in Fig. 5e). These are localized, however, in pericentric regions of repetitive DNA with few probes on the microarrays. Consequently, the apparent underreplication at these sites may not be biologically meaningful.

**Fig. 6:**
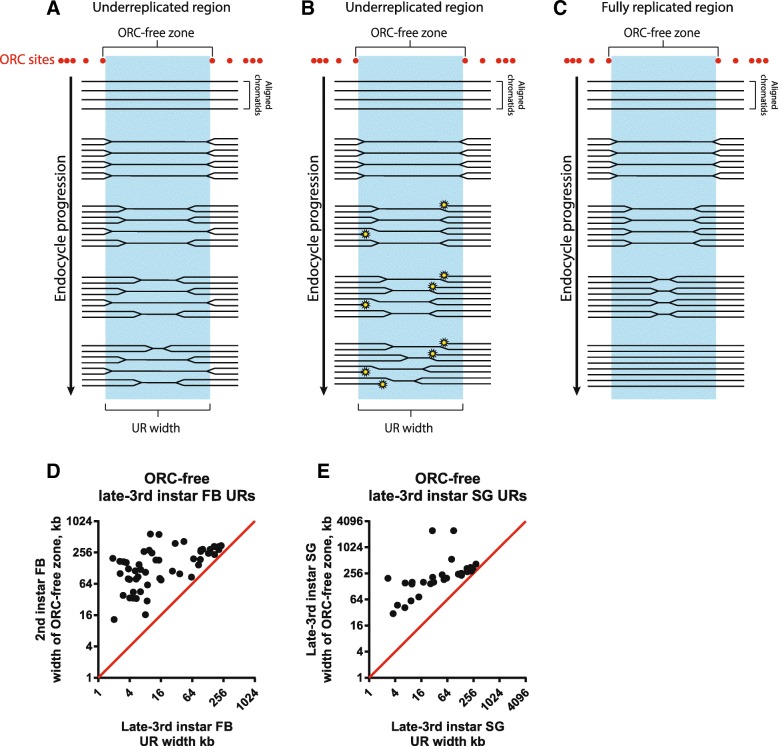
Relationship between ORC location and underreplication domains. **a**, **b** Models for the hypothesis of underreplication resulting from inhibition of replication fork progression. It is established that UR regions are largely repressed for ORC binding and thus replication initiation (ORC sites in red). Replication forks produced from flanking origins can cross into the potential URs. **a** Stochastic fork stalling would result in a gradient of decreased copy number, with the lowest relative copy number at the center of the UR region. **b** Destabilization of forks to generate double-strand breaks (yellow stars) could also account for the decreased copy number gradient. **c** At genomic regions where SUUR or other replication fork inhibitors do not act, replication forks from flanking origins can fully replicate the domain. **d**, **e** Relationship between the widths of ORC-free zones and the URs. **d** For each ORC-free UR in the larval fat body (FB), the nearest ORC binding sites to the left and right of the UR midpoint were determined and the distance between these sites was calculated as the ORC-free zone and compared to the width of the UR. **e** Widths of ORC-free zones were calculated using late-3rd instar salivary gland (SG) ORC2 ChIP-Seq data [8] relative to ORC-free larval SG UR widths. In all analyses, URs in pericentric heterochromatin regions were omitted

The above results suggested that underreplication may be actively regulated. Combining these with the finding that lack of ORC binding is insufficient to form URs, we hypothesized that tissue-specific control of replication fork progression is responsible for the properties of URs. The hypothesis is that replication forks generated at sites of ORC binding flanking the potential UR domains are subject to tissue-specific regulation such that their progress can be repressed or they can be destabilized (Fig. 6a, b). If this occurs stochastically, it would explain the gradient of reduced gene copy number. In tissues in which fork repression does not occur, replication forks from flanking origins can cross the ORC-free zones and ensure full copy number (Fig. 6c). Although one could propose that tissue-specific underreplication reflects differences in the length of S phase or timing of origin activation in different tissues, SUUR is required for underreplication of all of the URs in the salivary gland and in the fat body, including the newly-called URs presented in this study [15]. SUUR does not affect ORC binding or origin activity but does inhibit replication fork progression and stability [8, 17].

This hypothesis predicts that SUUR protein in the salivary gland should not be present in URs from other tissues that are replicated in the salivary gland. Unfortunately, it has not been possible to localize SUUR by ChIP-seq in the salivary gland (J. Nordman and T. Orr-Weaver, unpublished results). In the URs, however, a mark for the action of SUUR is the presence of the phosphorylated histone variant γH2Av (H2Ax in mammalian cells), indicative of double-strand breaks and dependent on SUUR function [17]. There are peaks of γH2Av in the salivary gland genome outside of the URs, but these are not dependent on SUUR [17]. By reanalyzing the genome-wide localization of γH2Av by ChIP-Seq [17], we found that 49% (35/71) of the salivary gland URs contain at least one γH2Av peak, with an average of 12.9 peaks per UR in these 35 URs. In contrast, only 12% (12/101) of the URs from other tissues that are fully replicated in the salivary gland contain a γH2Av peak, with an average of 8.5 peaks per UR in these 12 URs (Additional file 4: Figure S3D). These results are consistent with SUUR-induced double-strand breaks being present only in URs that are inhibited for replication. In addition, the observation that URs of increasing width have a higher level of underreplication (Fig. 4e) supports the proposal that underreplication results from stochastic failure of replication forks moving across the region from flanking ORCs.

As another test of the proposal that control of fork progression plays a crucial role in the tissue specificity of underreplication, we examined the position of adjacent ORC binding relative to the boundaries of the URs for the URs that are completely devoid of ORC. The width of the ORC-free region was quantified by measuring the distance between the closest ORC binding sites on either side of the center of the UR. When we examined the width of the URs in both the salivary gland and the fat body, we found that the width of the URs tended to increase with the width of the ORC-free zone (Fig. 6d, e). These results suggest that flanking ORCs limit the width of the UR, directing efficient initiation from these sites. In turn, the replication forks derived from these initiation events are required to replicate across the ORC-free domain. This hypothesis is supported by the observation that in the salivary gland the maximum fold underreplication increases with increasing width of the ORC-free zones, as replication forks would be required to traverse farther to ensure complete copy number (Additional file 7: Figure S6).

As a reciprocal test of the hypothesis that flanking ORCs define the URs by generating the replication forks whose progression and stability affect underreplication, we mapped the positions of low-density ORC domains in the salivary gland and examined whether they coincided with the locations of the URs. Low-density ORC regions were mapped by counting the number of ORC2 binding peaks across the genome in 150 kb windows, overlapping by 75 kb. Overlapping windows with no or one ORC2 binding peaks were merged to define a low-density ORC domain. The overlap between the low-density ORC domains and the URs is significant for all the URs in the genome (Jaccard index 0.22, Projection test *p* = 1.3e-04). The coincidence of these domains is particularly notable for the URs on chromosomes *2L* and *3L*, those that exhibit the most extensive underreplication (*2L* Jaccard index 0.51, Projection test *p* = 1.2e-07; *3L* Jaccard index 0.40, Projection test *p* = 1.8e-06) (Additional file 8: Figure S7). This is consistent with the boundaries of the URs being dictated by the positions of flanking ORC binding, also inferring that adjacent origins generate the replication forks responsible for differential DNA replication.

## Discussion

These results highlight the high degree of developmental control of DNA replication and URs. Even using a statistical method that calls a change in copy number of only 1.2 fold as significant, the majority of URs show tissue specificity. By mapping the sites of ORC binding in the larval fat body, permitting a comparison with the salivary gland, we found that there is tissue-specific regulation of the sites of ORC localization. In contrast, genomic regions with low density of ORC binding are constitutive across tissue and cell types. Importantly, these domains nearly devoid of ORC can be fully replicated in a tissue, even if they extend hundreds of kilobase pairs. The analyses presented here combined with previous studies on the replication fork inhibitor protein SUUR indicate that active tissue-specific control of replication forks is a major determinant of differential DNA replication. This insight is relevant to human cells, because it shows that when considering mechanisms that can give rise to genomic regions vulnerable to breakage after replication stress, such as Common Chromosome Fragile Sites [30], in addition to regulation of replication initiation it is important to recognize the critical role that developmental control of replication fork progression can play in genome stability.

The sites where ORC binds show extensive tissue specificity. Nearly a third of ORC binding sites are unique to the fat body. In comparison to diploid cells in culture, the polytene salivary gland and fat body share more ORC binding sites, as two thirds of ORC binding sites in the fat body are also sites where ORC is bound in the salivary gland. Thus, ORC positioning may be affected in part by either the endocycle or tissue differentiation. Identification of ORC binding sites in other differentiated Drosophila tissues will provide further insights into this regulation. There is a marked correlation with ORC being bound within 1 kb of active transcription sites in the fat body, with 95% of ORC sites corresponding to expressed genes. Although this adds to the link between ORC binding and transcriptional control regions observed in Drosophila tissues, cell lines, and human cell lines, it is important that, as in the salivary gland, transcriptional activity is not sufficient to account for the tissue-specific sites of ORC binding.

The ORC binding analyses reveal genomic regions in which ORC localization is inhibited in a constitutive way across many tissues. These domains lie within TADs, but it remains to be determined how genome organization impacts ORC binding. The results with the larval fat body show that an inhibitory effect on ORC binding and transcription do not have to be linked. In addition, defining the causal consequences of the lack of ORC in these regions will be important. Their constitutive nature across tissues argues for a function for zones inhibitory for replication. This is further suggested by late replicating regions being conserved not only between tissues but also between species [31]. It suggests that even in diploid cells inhibiting replication so that it occurs late in S phase is important. Contributing to this inhibition and thus timing of replication domains in S phase may be the crucial role of proteins like SUUR. A reduction in ORC binding density in late replicating genome regions was observed also in human cells [7], thus this may be a conserved feature of the organization of metazoan genomes.

It was unexpected and is significant that as DNA replication ceases in the fat body cells in the 3rd-instar larval stage, ORC is cleared from the chromatin. This was observed both by immunofluorescence and ChIP analysis. The adult fat body is formed from persisting larval fat body cells, suggesting that because of removal in the last larval stage, ORC would have to be reloaded to permit subsequent polyploidization in the adult fat body. It is puzzling why this occurs; perhaps ORC is loaded to distinct sites in the adult cells.

We propose that replication of low-density ORC domains in some but not all endocycling tissues reflects active inhibition of replication fork progression when these regions are underreplicated. This explanation is supported by six lines of evidence: 1) all of the URs are dependent on SUUR, a protein that inhibits replication fork progression and generates double-strand breaks but has no demonstrated effect on ORC localization or replication initiation; 2) the lack of correlation between ploidy and the number of URs or their fold underreplication; 3) the absence of γH2Av in the salivary gland in regions replicated in the salivary gland but underreplicated in other tissues; 4) the boundaries of the URs lie within the width of the ORC-free zone; 5) URs of increasing width have an increasing width of the ORC-free zone and more reduction of copy number; and 6) the coincidence between the positions of low-density ORC regions in the genome and the sites of URs. A critical role of inhibition of fork progression in underreplication is supported also by the recent finding that the E2F/DP transcription factor represses transcription of the checkpoint sensor *dATM* [32]. In *dDP* mutants, elevation of dATM is associated with decreased underreplication, suggesting that excessive dATM may signal repair of stalled forks that normally lead to underreplication.

An intriguing implication of the tissue and region-specific control of replication fork progression is the level of regulation that must be exerted on replication fork proteins. Thus it will be critical to identify other proteins in addition to SUUR involved in this regulation, as well as to define the mechanisms that promote their association with and inhibition of replication forks at specific regions in specific tissues. One insight comes from a recent study demonstrating a role for histone H1 in controlling the association of SUUR with chromatin dynamically during S phase [33]. In addition, delineating the role of dATM at replication forks in URs will be an important future goal.

It has been proposed that polytene tissues in Drosophila can replicate their DNA without ORC [34], and human cell lines disrupted for ORC1 and ORC2 are viable and undergo DNA replication [35]. Thus it is possible that the full replication observed in the fat body or salivary gland for ORC-free regions occurs through ORC-independent initiation of DNA replication within the domain rather than by tissue-specific control of fork progression. The properties of SUUR and the double-strand breaks generated across regions in which it impedes fork progression argue against this possibility. Because all of these URs require SUUR, then when they are fully replicated in the fat body or the salivary gland either: 1) SUUR must not be present or not active at the region; or 2) SUUR could be active but ORC-independent initiation permits full replication. By immunofluorescence SUUR is not detectable at polytene bands in the salivary gland chromosomes containing URs specifically underreplicated in the fat body but replicated in the salivary gland [16, 36]. The absence of γH2Av also is consistent with SUUR not being active. In sum, these observations support the idea that replication of the ORC-free zones is due to the absence of SUUR inhibition of fork progression permitting forks generated by flanking origins to replicate across the domain.

## Conclusions

Genomic organization is crucial in the distribution of ORC binding, both in conferring tissue-specificity of where ORC localizes and in establishing regions of the genome that are nearly devoid of ORC in endocycling cells and have reduced ORC density in diploid cells. These regions of low origin density generate the potential for late replication in diploid cells and underreplication in endocycling cells. This parallels the finding in human cell lines that 73% of regions containing Chromosome Fragile Sites and commonly deleted in cancer cells overlap ORC-poor domains [7]. The results presented here indicate that replication defects mediated by inhibition of replication initiation can be augmented by negative control of replication fork progression, leading to tissue specificity of whether an ORC-free domain is under or fully replicated. This highlights a critical role for the active regulation of replication fork progression in metazoans in controlling genome stability.

## Methods

### Comparative genomic hybridization

Midgut and Malpighian tubules were dissected from wild-type *OrR* adult females 0–6 h after eclosion. Genomic DNA was isolated from these tissues and from 0 to 6 h diploid control embryos as described [37]. Genomic DNA from experimental and control tissues was differentially labeled using the Invitrogen BioPrime Total for Agilent aCGH kit according to the manufacturer’s protocol. Labeled DNA was hybridized to tilling arrays containing probes on average every 1500 bp, 250 bp, or 125 bp spanning the Drosophila genome and washed as recommended by Agilent protocol. Array intensities were LOESS normalized and log_2_ values obtained using the Ringo package in R [38]. Biological replicates were performed for each tissue.

In one method, underreplication was defined as a two-fold reduction in copy number in regions of 10 kb or greater in both biological replicates and determined using the MA2C peak calling analysis software [39]. In the new method, by which the URs analyzed in this paper were defined, for each sample, the distribution of all log_2_ ratios was determined, and the smoothed probes were then scaled, setting the peak of the distribution to 0. Next, we used the distribution to set the copy number threshold for under replication. In more detail, the distribution was split at its peak, and the over replication side of the curve was treated as representative of the null distribution. The log_2_ ratio representing the 5th percentile of the null distribution was set as the copy number threshold (Z score of − 2, two-sided *p*-value of 0.05). Any probe exhibiting a log_2_ ratio less than this threshold was classified as underreplicated. Any region that contained at least 25/11/5 underreplicated probes in a row (for array designs of 1 M, 400 k, and 180 k probes, respectively) was defined as an underreplicated region. Underreplicated regions within 50 kb were then merged. For each pair of sample duplicates, the intersection of underreplicated regions for duplicate samples was defined as the high-confidence set of underreplicated regions.

### Ploidy quantification

Malpighian tubules were isolated from 0 to 6 h *OrR* adults and imaginal discs from 3rd-instar wandering larvae (a mixture of males and females) by manual dissection in Grace’s unsupplemented media. Tissues were fixed with 4% formaldehyde in PBS for 10 min, washed, and stained with 50 ng/mL DAPI for 10 min at room temperature. Tissues were mounted on slides and imaged on a Nikon Eclipse Ti epifluorescence microscope with a 60X oil-immersion objective using a Hamamatsu ORCA-ERA camera. DAPI intensities were measured for individual nuclei using the Nikon Elements Advanced Research software. DAPI intensities for adult Malpighian tubule nuclei were normalized to diploid larval imaginal disc nuclei to calculate ploidy.

### RNA-sequencing

*OrR* eggs were collected for 1 h and incubated at 25 °C. 67–68 or 91–92 h after egg laying (AEL), 30 male larvae were dissected for fat body in Ephrussi-Beadle Ringer (EBR) solution [40], and testes were removed manually. Fat body was homogenized in Trizol LS reagent using an electric homogenizer. 5μg total RNA was then poly-A selected and mRNA-seq libraries were prepared using the NEBNext mRNA Library Prep Reagent Set for Illumina (NEB) according to the manufacturer’s instructions. Samples were prepared in biological replicate, and 40 nt paired-end reads were generated on the Illumina HiSeq2000. Reads were aligned to the dm3 genome using TopHat [41] and FPKM values were calculated with Cufflinks [42]. A high level of reproducibility was observed between biological replicates (minimum R^2^ > 0.86, Pearson’s correlation). Thus, FPKM values were averaged between biological replicates for analysis.

Adult midgut and Malpighian tubules mRNA-seq libraries were prepared and sent for high-throughput sequencing in collaboration with Brenton Graveley (UConn Health Center) as part of the modENCODE project.

### ChIP-sequencing

*OrR* eggs were collected as described above. 120 male 67-68 h AEL, 60 male 91-92 h AEL, or 60 3rd-instar wandering larvae were isolated in EBR, and cuticles were pulled back to expose all tissues. Whole, flayed larvae were fixed in 2% formaldehyde for 15mins at room temperature. The fat body then was isolated from the larvae in ChIP Lysis Buffer (50 mM HEPES pH 7.5, 140 mM NaCl, 1 mM EDTA, 1% Triton X-100, 0.1% Na-Deoxycholate), and testes were removed manually. The fat body was dounce homogenized using a glass tight pestle to disrupt the tissue. Chromatin was fragmented by sonication in a Biorupter300 (Diagenode) at 4 °C for 30 cycles of 30s on, 30s off at maximum power. Supernatants were incubated with 1:250 anti-ORC2 serum overnight at 4 °C. ORC2 antibodies were pulled down with a 50:50 mixture of Protein A and Protein G-coupled Dynabeads (ThermoFisher Scientific). Crosslinks were reversed overnight at 65 °C. DNA was treated with Proteinase K and RNase A before purification by phenol-chloroform extraction. Libraries were generated using the NEBNext Ultra DNA Library Prep Kit for Illumina (NEB) according to the manufacturer’s instructions. Samples were prepared in biological replicate, and 40 nt paired-end reads were generated on the Illumina HiSeq2000. Reads were aligned to the dm3 genome with Bowtie2 using default settings [43]. Peaks were called for individual replicates using MACS2 [44] with q-value< 0.05 and normalizing to input. High-confidence ORC2 peaks common to both biological replicates were then identified using the irreproducible discovery rate (IDR) metric [24] using an IDR < 0.1 Peaks within 1 kb were treated as overlapping.

### EdU-labeling and immunofluorescence

*OrR* eggs were collected as above. 67–68 AEL, 91–92 AEL, and 3rd-instar wandering larvae were isolated in EBR and cuticles were pulled back to expose all larval tissues. Whole larvae were labeled with 50 μM EdU for 30 min at room temperature. Labeled larvae were then fixed in 8% formaldehyde for 5 min at room temperature. Larvae were permeablized in 1X PBS + 1% Triton-X100 for 2 h at room temperature and blocked with 1X PBS + 0.3% Triton-X100 + 1% BSA + 2% normal goat serum. Larvae then were incubated with 1:2500 anti-ORC2 serum overnight at 4 °C. ORC antibodies were detected with FITC-conjugated anti-rabbit antibody at a 1:200 dilution. EdU was detected by Alexaflour555-azide using standard Click-iT chemistry (ThermoFisher). Finally, fat body tissue was isolated from labeled larvae, mounted on slides, and imaged on a Nikon Eclipse Ti epifluorescence microscope with the 10X objective using a Hamamatsu ORCA-ERA camera.

### Analysis of UR width and depth

For each UR identified in each tissue, the width of each UR was doubled, followed by LOESS smoothing at 10-nt resolution. The minimal log2 ratio within the smoothened domain was set as the maximal fold underreplication value of the UR. From this minimum within the region, the probes exhibiting half the minimal log2 ratio value to the left and right were defined as the left half-minimum and right half-minimum, respectively. The distance between the left and right half-minimum probes was defined as the half-min width.

### Analysis of ORC sites near URs

For each UR identified in the late-3rd instar wandering larval salivary gland and the 67-68 h AEL larval fat body, the nearest ORC2 site to the left of the UR midpoint was determined. Next, the nearest ORC2 site to the right of the UR midpoint was determined. The distance between these two ORC2 sites was calculated as the width of the ORC-free zone.

### Mapping low-density ORC regions

Late-3rd instar wandering larval salivary gland ORC2 ChIP-seq peaks [8] were analyzed by counting their number in 150 kb windows, overlapping by 75 kb. Windows with 0 or 1 ORC2 peaks on the canonical chromosomes were identified and overlapping windows were merged. These windows, denoted as low ORC density regions, were compared to the combined set of URs from the five tissues characterized in this study using both the absence/presence of overlap and the width of overlap, the latter with GenometricCorr [45] using 10,000 permutations. The magnitude and statistical significance of the overlap was calculated using the Jaccard index and the projection test, respectively. The overlap between the low-density ORC domains and all of the URs in the genome was calculated with GenometricCorr: KS test *p* < 0.005; Jaccard index: 0.22; Projection test: *p* = 1.3e-04.

## Additional files

Additional file 1: **Figure S1.** Differentiation of the adult Malpighian tubules and the adult midgut. A) The Malpighian tissue persists into adulthood. B) The larval midgut is destroyed during pupation and is built anew in the adult from diploid progenitors. C) Ploidy values of individual nuclei from adult Malpighian tubules compared to nuclei from the larval midgut (larval midgut ploidy data from [15]). (PDF 848 kb)

Additional file 2: **Figure S2.** aCGH profiles of the adult Malpighian tissue (blue) compared to the aCGH profile of the larval midgut tissue (black) and the adult midgut tissue (red). Bars below aCGH profiles represent regions of underreplication called by the statistical method. Chromosome coordinates from the dm3 genome are shown. (PDF 1175 kb)

Additional file 3: **Table S1.** Genomic coordinates of underreplicated regions in endocycling larval and adult tissues. The sequence coordinates and cytological positions are shown for the regions called as underreplicated in the five tissues analyzed. A (+) indicates that the region was significantly underreplicated. The last column shows the size of the underreplicated domain. (PDF 312 kb)

Additional file 4: **Figure S3.** Comparison of ORC binding in the larval salivary gland (SG) with underreplication. A) aCGH profile of late-3rd-instar larval salivary gland. UR regions in the late-3rd-instar larval salivary gland are denoted as black boxes and are highlighted by green shading. Regions that are underreplicated in other larval tissues but are fully replicated in late-3rd-instar larval salivary gland are denoted as red boxes. The peak summit locations of ORC2 from the salivary gland relative to dm3 genome coordinates are shown (the aCGH and ORC ChIP data are from [8]). B) Comparison of the number of salivary gland ORC2 binding sites per 100 kb within the late-3rd-instar larval salivary gland UR regions relative to fully replicated regions. *p < 10^–5^. C) Comparison of the number of salivary gland ORC2 binding sites per 100 kb within regions that are underreplicated in other larval tissues but are fully replicated in late-3rd-instar larval salivary gland relative to fully replicated regions. *p < 10^–5^. D) Highlighted region from *chr2L* containing three URs from the late-3rd-instar larval salivary gland (black boxes and green shading) and two URs fully replicated in the salivary gland but underreplicated in at least one other larval tissue (red boxes). Late-3rd-instar larval salivary gland ORC2 peaks are shown in blue and γH2Av peaks in orange. (PDF 948 kb)

Additional file 5: **Figure S4.** Analysis of ORC-free regions in endocycling tissues and cultured cells. A) Copy number and UR profiles of *chr2L* of late-3rd-instar larval salivary gland (3WL SG) and late-3rd-instar larval fat body (3WL FB) overlayed with ORC2 ChIP-seq peaks from endocycling tissues (3WL SG and 3WL FB) and from cultured diploid cells (BG3, Kc, S2; data from [6]). B) Magnified region of *chr2L* from (A). C) Genome-wide analysis of ORC2 peaks within the combined UR domains (across all five endocycling tissues) from endocycling tissues and cultured diploid cells. (PDF 964 kb)

Additional file 6: **Figure S5.** Comparison of UR regions with topologically associated domains (TADs). A) aCGH plot of late-3rd-instar larval salivary gland (data from [8]) of *chr2L*. Red boxes represent UR regions in the larval salivary gland. Green boxes represent the combined UR regions across all five tissues examined in this study. Orange boxes represent the larval salivary gland TADs from [26]. B) Venn diagram showing the extent of overlap between the UR regions identified in the larval salivary gland in this study and the salivary gland TADs. C) Venn diagram showing the extent of overlap between all UR regions identified the five tissues examined in this study and the TADs. To assess the statistical significance of the overlap between the URs and the salivary gland TADs, genomic regions of the same number and widths of the actual URs were selected at random 10^6^ times and compared to the TAD locations. For each iteration, the number of overlaps of those genomic regions with the TAD domains were determined and a distribution of number of overlaps for all the iterations was plotted. The *p*-value for the number of overlaps of the actual URs with the TADs was then determined from that distribution. (PDF 844 kb)

Additional file 7: **Figure S6.** Relationship between ORC localization and extent of underreplication. A) Widths of the ORC-free zones from URs in the larval fat body compared to maximum fold underreplication. B) Widths of the ORC-free zones from URs in the larval salivary gland compared to maximum fold underreplication. All plots are displayed with log2 axes. (PDF 803 kb)

Additional file 8: **Figure S7.** Analysis of regions of low ORC density in the larval salivary gland on *chr2L* and *chr3L*. ORC2 ChIP-Seq peaks from late-3rd-instar larval salivary gland [8] are depicted in blue. ORC2 peaks were analyzed by counting their number in 150 kb windows with 75 kb overlaps. Windows containing 0 or 1 ORC2 peaks were identified. From these windows, overlapping windows were merged and depicted as black bars with the number of ORC2 peaks contained within each window noted below. All URs combined from the five tissues characterized in this study are shown in orange. Late-3rd-instar larval salivary gland URs containing ORC are depicted as green bars with the number of ORC2 peaks within each UR shown below. Late-3rd-instar larval salivary gland URs that do not contain ORC2 peaks are shown as red bars. (PDF 844 kb)

## Abbreviations

aCGH: Array comparative genome hybridization
ChIP-Seq: Chromatin immunoprecipitation with high throughput DNA sequencing
ORC: Origin Recognition Complex
RNA-Seq: High throughput RNA sequencing
TAD: Topologically Associated Domain
URs: Underreplicated regions
