# Supplementary material for: Dynamic changes in ORC localization and replication fork progression during tissue differentiation
Source: BMC Genomics. 2018 Aug 22;19:623. doi: 10.1186/s12864-018-4992-3 (PMC6103881; doi:10.1186/s12864-018-4992-3)
Supplement: Supplementary file 1 — Figure S1. Differentiation of the adult Malpighian tubules and the adult midgut. A) The Malpighian tissue persists into adulthood. B) The larval midgut is destroyed during pupation and is built anew in the adult from diploid progenitors. C) Ploidy values of individual nuclei from adult Malpighian tubules compared to nuclei from the larval midgut (larval midgut ploidy data from [15]). (PDF 848 kb) [file 12864_2018_4992_MOESM1_ESM.pdf]

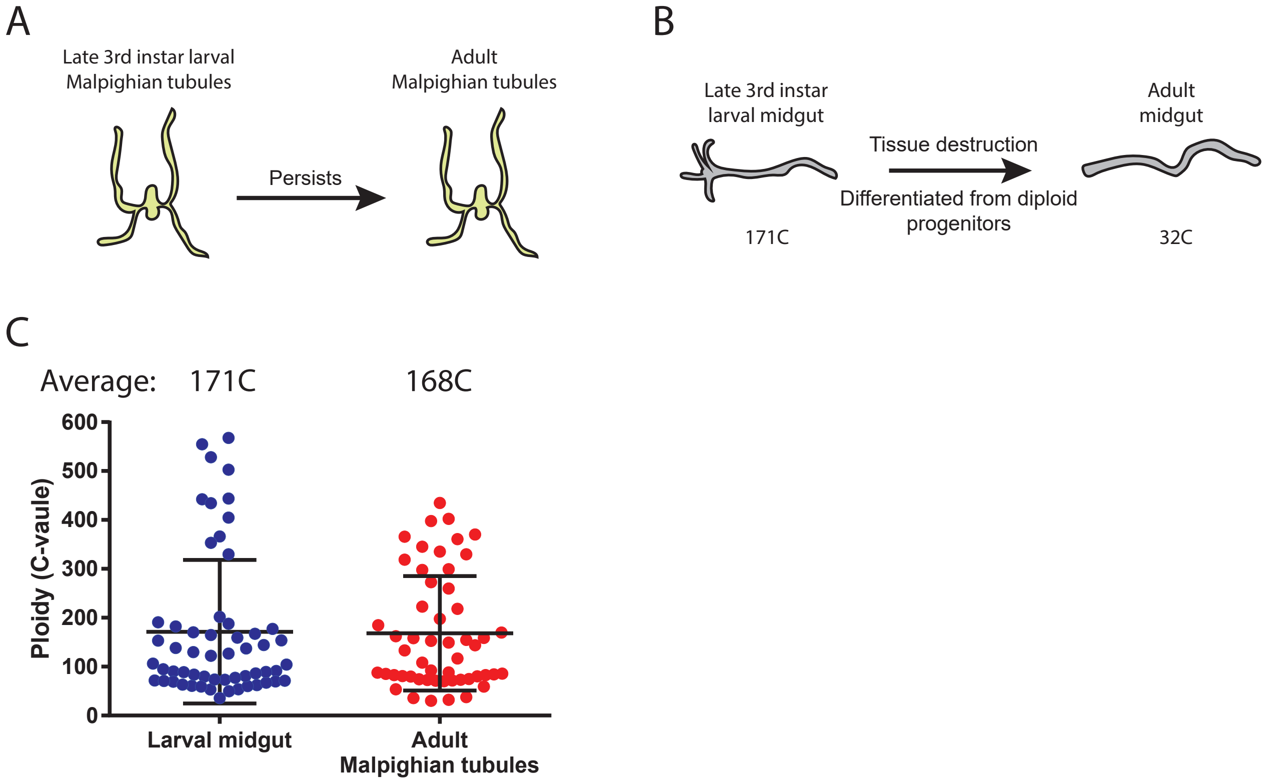
A
Late 3rd instar larval
Malpighian tubules
Persists
Adult
Malpighian tubules
B
Late 3rd instar
larval midgut
Tissue destruction
Differentiated from diploid
progenitors
Adult
midgut
171C
32C
C
Average: 171C
168C
Ploidy (C-vaule)
600
500
400
300
200
100
0
Larval midgut
Adult
Malpighian tubules
