# Supplementary figures and images for: Dynamic changes in ORC localization and replication fork progression during tissue differentiation
Source: BMC Genomics. 2018 Aug 22;19:623. doi: 10.1186/s12864-018-4992-3 (PMC6103881; doi:10.1186/s12864-018-4992-3)

*chrX*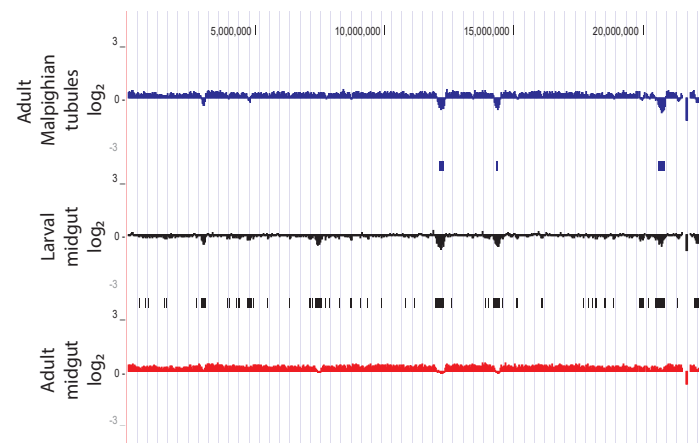*chr2L*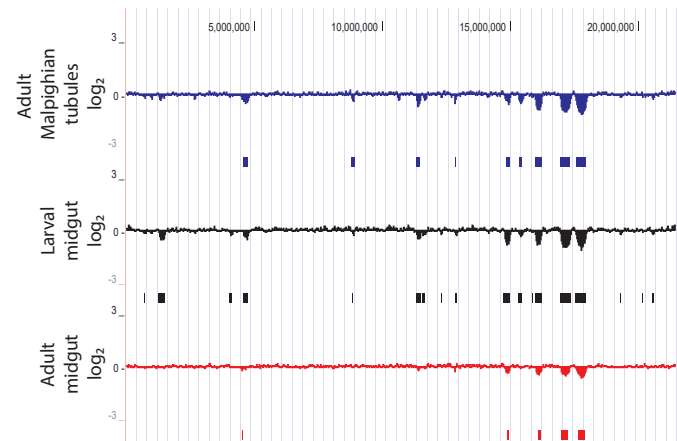*chr2R*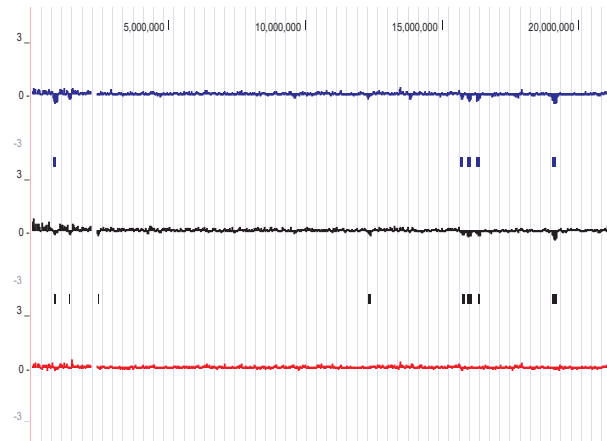*chr3L*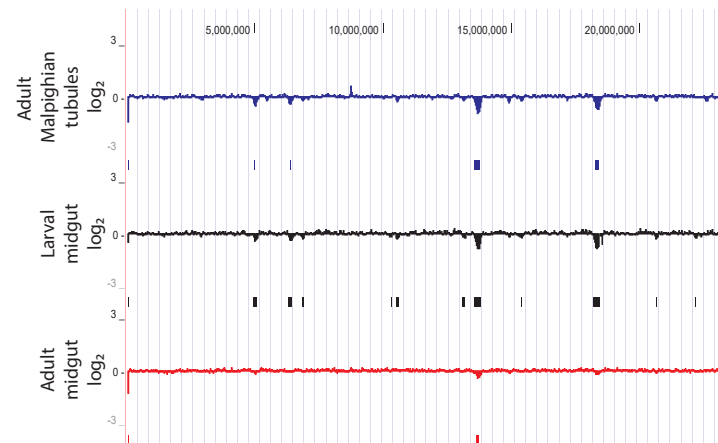*chr3R*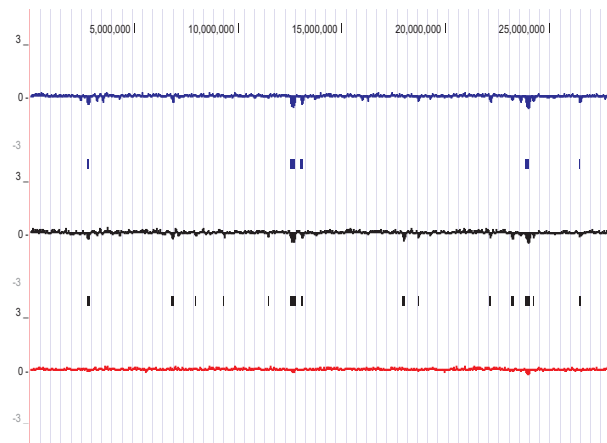

Supplement: Supplementary file 2 — Figure S2. aCGH profiles of the adult Malpighian tissue (blue) compared to the aCGH profile of the larval midgut tissue (black) and the adult midgut tissue (red). Bars below aCGH profiles represent regions of underreplication called by the statistical method. Chromosome coordinates from the dm3 genome are shown. (PDF 1175 kb) [file 12864_2018_4992_MOESM2_ESM.pdf]

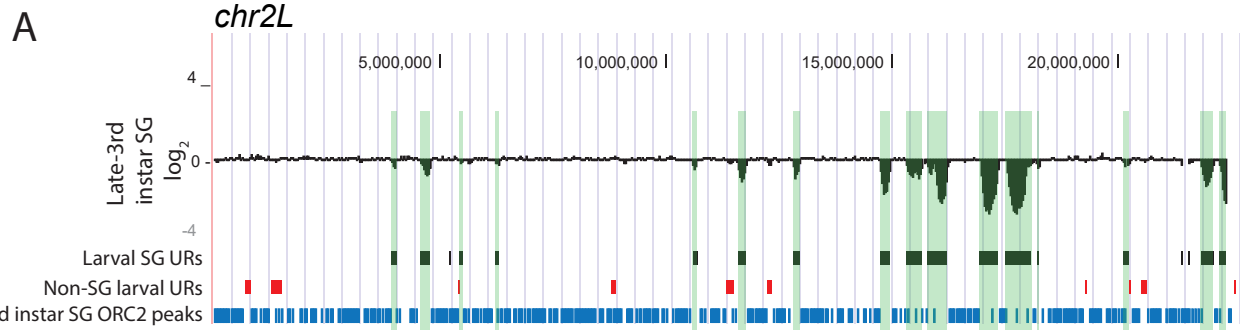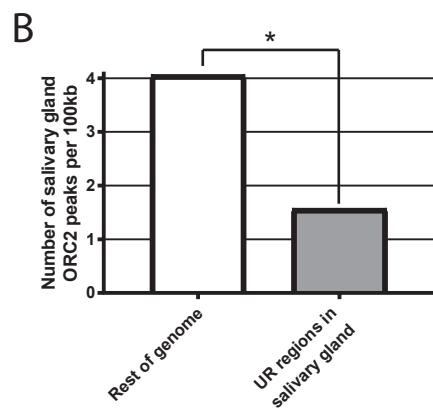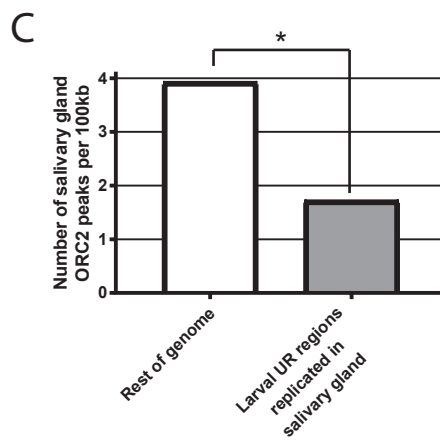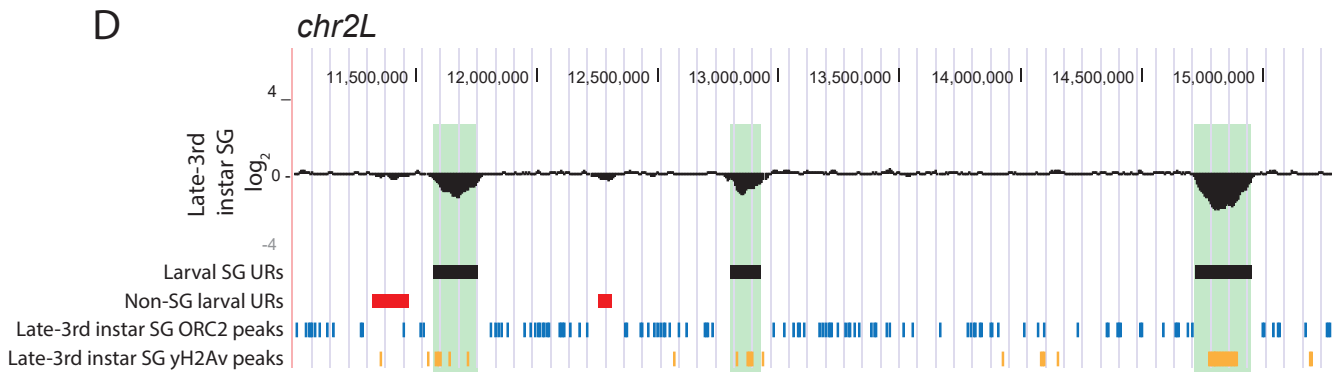

Supplement: Supplementary file 4 — Figure S3. Comparison of ORC binding in the larval salivary gland (SG) with underreplication. A) aCGH profile of late-3rd-instar larval salivary gland. UR regions in the late-3rd-instar larval salivary gland are denoted as black boxes and are highlighted by green shading. Regions that are underreplicated in other larval tissues but are fully replicated in late-3rd-instar larval salivary gland are denoted as red boxes. The peak summit locations of ORC2 from the salivary gland relative to dm3 genome coordinates are shown (the aCGH and ORC ChIP data are from [8]). B) Comparison of the number of salivary gland ORC2 binding sites per 100 kb within the late-3rd-instar larval salivary gland UR regions relative to fully replicated regions. *p < 10–5. C) Comparison of the number of salivary gland ORC2 binding sites per 100 kb within regions that are underreplicated in other larval tissues but are fully replicated in late-3rd-instar larval salivary gland relative to fully replicated regions. *p < 10–5. D) Highlighted region from chr2L containing three URs from the late-3rd-instar larval salivary gland (black boxes and green shading) and two URs fully replicated in the salivary gland but underreplicated in at least one other larval tissue (red boxes). Late-3rd-instar larval salivary gland ORC2 peaks are shown in blue and γH2Av peaks in orange. (PDF 948 kb) [file 12864_2018_4992_MOESM4_ESM.pdf]

A

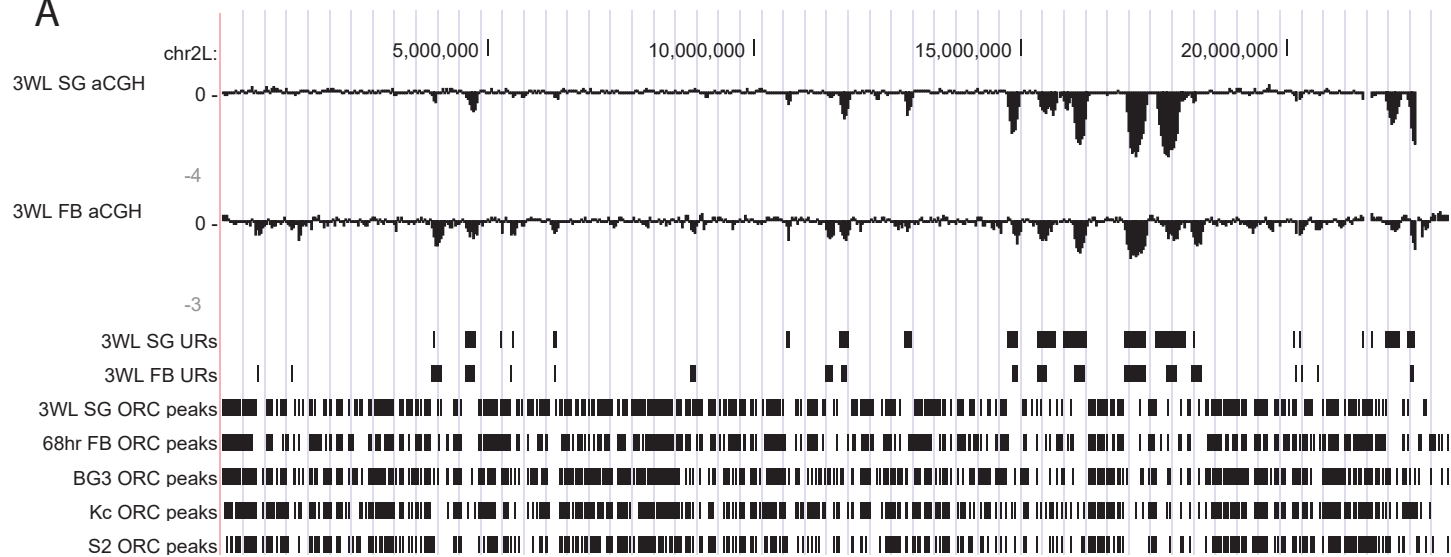

B

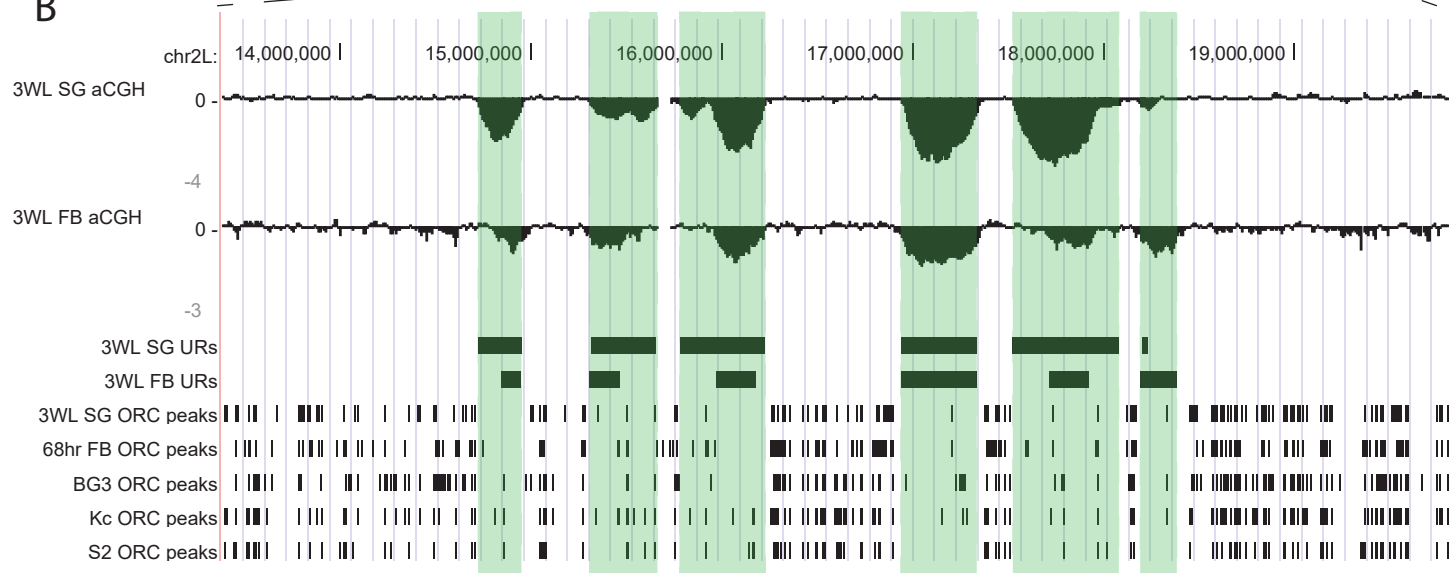

C

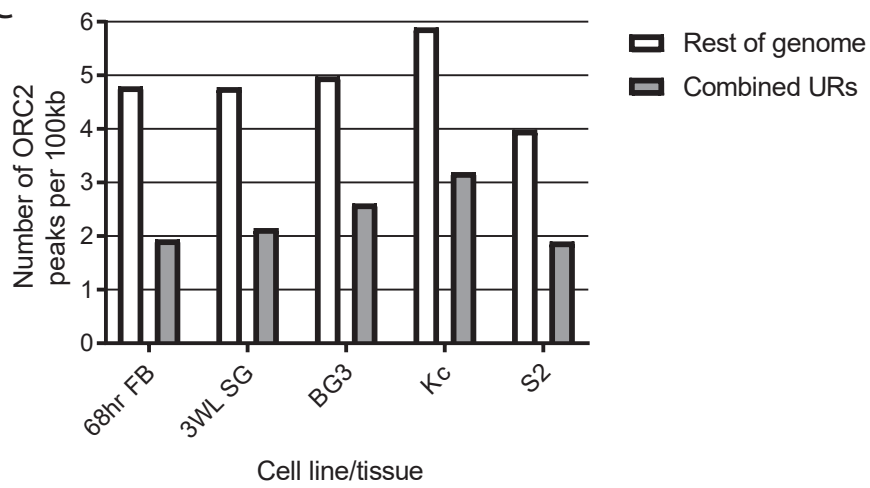

Supplement: Supplementary file 5 — Figure S4. Analysis of ORC-free regions in endocycling tissues and cultured cells. A) Copy number and UR profiles of chr2L of late-3rd-instar larval salivary gland (3WL SG) and late-3rd-instar larval fat body (3WL FB) overlayed with ORC2 ChIP-seq peaks from endocycling tissues (3WL SG and 3WL FB) and from cultured diploid cells (BG3, Kc, S2; data from [6]). B) Magnified region of chr2L from (A). C) Genome-wide analysis of ORC2 peaks within the combined UR domains (across all five endocycling tissues) from endocycling tissues and cultured diploid cells. (PDF 964 kb) [file 12864_2018_4992_MOESM5_ESM.pdf]

A

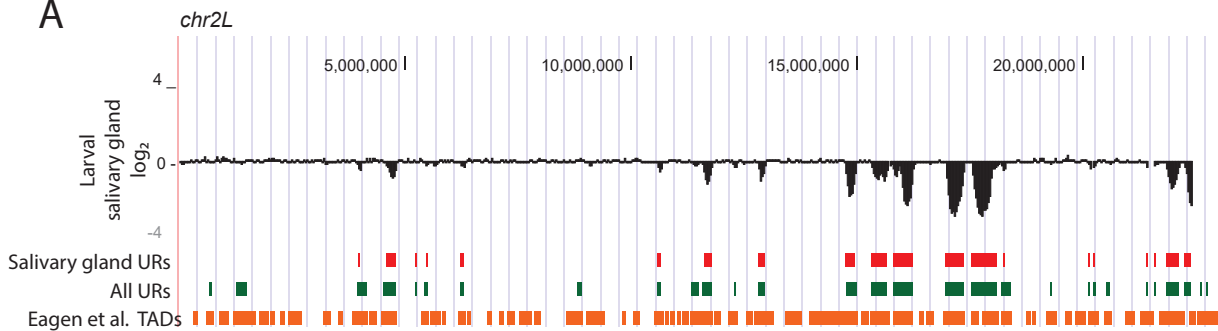

B

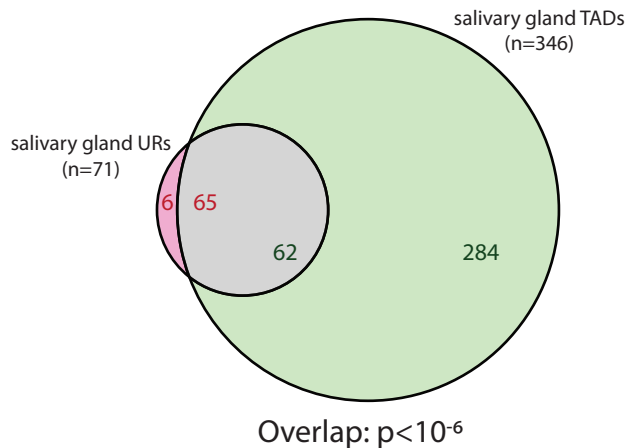

C

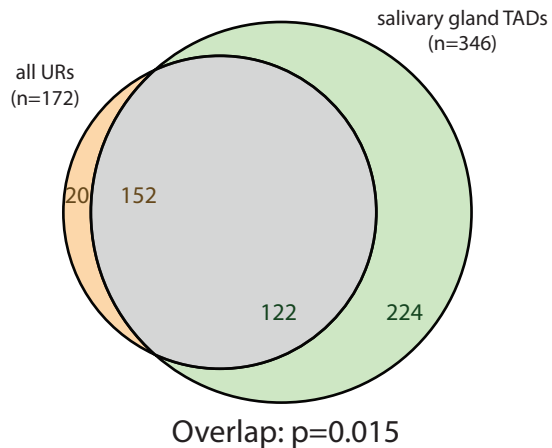

Supplement: Supplementary file 6 — Figure S5. Comparison of UR regions with topologically associated domains (TADs). A) aCGH plot of late-3rd-instar larval salivary gland (data from [8]) of chr2L. Red boxes represent UR regions in the larval salivary gland. Green boxes represent the combined UR regions across all five tissues examined in this study. Orange boxes represent the larval salivary gland TADs from [26]. B) Venn diagram showing the extent of overlap between the UR regions identified in the larval salivary gland in this study and the salivary gland TADs. C) Venn diagram showing the extent of overlap between all UR regions identified the five tissues examined in this study and the TADs. To assess the statistical significance of the overlap between the URs and the salivary gland TADs, genomic regions of the same number and widths of the actual URs were selected at random 106 times and compared to the TAD locations. For each iteration, the number of overlaps of those genomic regions with the TAD domains were determined and a distribution of number of overlaps for all the iterations was plotted. The p-value for the number of overlaps of the actual URs with the TADs was then determined from that distribution. (PDF 844 kb) [file 12864_2018_4992_MOESM6_ESM.pdf]

A

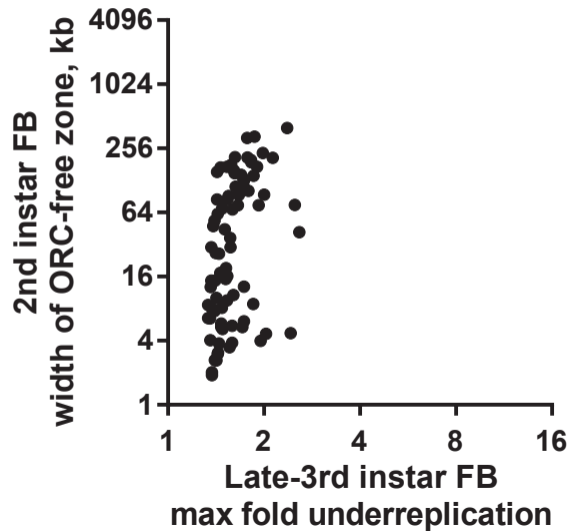

B

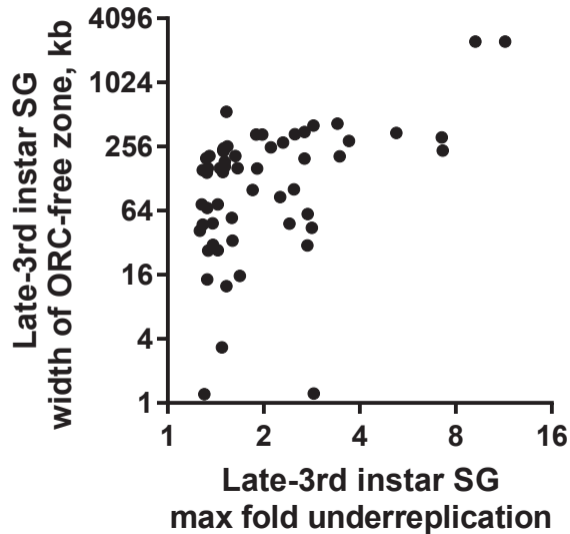

Supplement: Supplementary file 7 — Figure S6. Relationship between ORC localization and extent of underreplication. A) Widths of the ORC-free zones from URs in the larval fat body compared to maximum fold underreplication. B) Widths of the ORC-free zones from URs in the larval salivary gland compared to maximum fold underreplication. All plots are displayed with log2 axes. (PDF 803 kb) [file 12864_2018_4992_MOESM7_ESM.pdf]

## *chr2L*

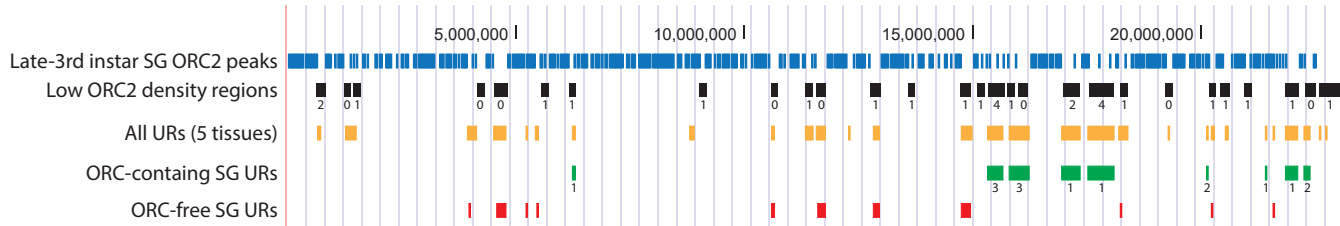

## *chr3L*

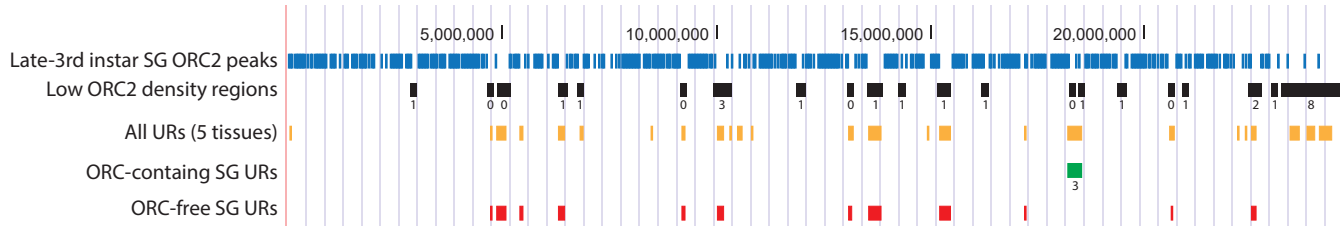

Supplement: Supplementary file 8 — Figure S7. Analysis of regions of low ORC density in the larval salivary gland on chr2L and chr3L. ORC2 ChIP-Seq peaks from late-3rd-instar larval salivary gland [8] are depicted in blue. ORC2 peaks were analyzed by counting their number in 150 kb windows with 75 kb overlaps. Windows containing 0 or 1 ORC2 peaks were identified. From these windows, overlapping windows were merged and depicted as black bars with the number of ORC2 peaks contained within each window noted below. All URs combined from the five tissues characterized in this study are shown in orange. Late-3rd-instar larval salivary gland URs containing ORC are depicted as green bars with the number of ORC2 peaks within each UR shown below. Late-3rd-instar larval salivary gland URs that do not contain ORC2 peaks are shown as red bars. (PDF 844 kb) [file 12864_2018_4992_MOESM8_ESM.pdf]
