# Supplementary material for: Dynamic changes in ORC localization and replication fork progression during tissue differentiation
Source: BMC Genomics. 2018 Aug 22;19:623. doi: 10.1186/s12864-018-4992-3 (PMC6103881; doi:10.1186/s12864-018-4992-3)
Supplement: Supplementary file 3 — Table S1. Genomic coordinates of underreplicated regions in endocycling larval and adult tissues. The sequence coordinates and cytological positions are shown for the regions called as underreplicated in the five tissues analyzed. A (+) indicates that the region was significantly underreplicated. The last column shows the size of the underreplicated domain. (PDF 312 kb) [file 12864_2018_4992_MOESM3_ESM.pdf]

Table S1. Genomic coordinates of underreplicated regions in endocycling larval and adult tissues.

Chromosome	Start	End	Cytological position	Larval fat body	Larval midgut	Larval salivary gland	Adult midgut	Adult Malpighian tubules	Size (bp)
chrX	8342	17610	*1A*	-	-	+	-	-	9268
chrX	453194	457218	*1B*	-	+	-	-	-	4024
chrX	695362	701602	*1C*	-	+	-	-	-	6240
chrX	752258	756022	*1D*	+	-	-	-	-	3764
chrX	839162	841512	*1D*	-	+	-	-	-	2350
chrX	1418532	1454352	*2B*	-	+	-	-	-	35820
chrX	1504862	1521882	*2B*	-	+	-	-	-	17020
chrX	2323610	2328985	*3A*	+	-	-	-	-	5375
chrX	2662628	2686382	*3B*	+	+	-	-	-	23754
chrX	2727804	2758072	*3C*	+	-	-	-	-	30268
chrX	2849012	3018692	*3C*	+	+	+	-	-	169680
chrX	3628660	3631298	*3E*	+	-	-	-	-	2638
chrX	3846340	3861370	*3F-4A*	-	+	-	-	-	15030
chrX	3877402	3939316	*4A*	+	-	-	-	-	61914
chrX	3945252	3963822	*4A*	-	+	-	-	-	18570
chrX	4206784	4213952	*4B*	-	+	-	-	-	7168
chrX	4306782	4390342	*4C*	+	+	-	-	-	83560
chrX	4631148	4791054	*4C-4D*	+	+	-	-	-	159906
chrX	4864634	4871487	*4E*	-	+	-	-	-	6853
chrX	4982244	5135836	*4E-4F*	+	-	-	-	-	153592
chrX	5422870	5443702	*5A*	-	+	-	-	-	20832
chrX	5469662	5479182	*5A*	+	-	-	-	-	9520
chrX	5910952	5923822	*5D*	+	-	-	-	-	12870
chrX	6283922	6310812	*5F-6A*	+	+	-	-	-	26890
chrX	7043648	7163710	*6F-7A*	+	+	-	-	-	120062
chrX	7194053	7198317	*7B*	-	+	-	-	-	4264
chrX	7259806	7533146	*7B*	+	+	-	-	-	273340
chrX	7655262	7703084	*7B-7C*	+	+	-	-	-	47822
chrX	7822820	7826142	*7C*	-	+	-	-	-	3322
chrX	7919216	7923032	*7D*	+	-	-	-	-	3816
chrX	8199550	8204718	*7E*	-	+	-	-	-	5168
chrX	8638618	8701082	*8A-8B*	+	+	-	-	-	62464
chrX	9013688	9023684	*8C*	-	+	-	-	-	9996
chrX	9293782	9368660	*8D-8E*	+	+	-	-	-	74878
chrX	9630248	9638304	*9A*	+	-	-	-	-	8056
chrX	9838120	9847680	*9A*	-	+	-	-	-	9560
chrX	10750822	10753082	*9F*	-	+	-	-	-	2260
chrX	11123012	11126332	*10B*	-	+	-	-	-	3320
chrX	11958399	12297968	*11A*	+	+	+	-	+	339569
chrX	12557845	12566582	*11B*	-	+	-	-	-	8737
chrX	13862604	13866792	*12D*	-	+	-	-	-	4188
chrX	13955963	13983242	*12D-12E*	-	-	+	-	-	27279
chrX	14009548	14037662	*12E*	-	+	-	-	-	28114
chrX	14184152	14466640	*12E*	+	+	+	-	+	282488
chrX	14523404	14563550	*12F*	-	+	-	-	-	40146
chrX	14869642	14871542	*13A*	+	-	-	-	-	1900
chrX	15066072	15152635	*13B*	+	+	-	-	-	86563
chrX	16045416	16086482	*14B*	-	+	-	-	-	41066
chrX	16125270	16128732	*14B*	+	-	-	-	-	3462

chrX	17241800	17247590	*16A*	+	-	-	-	-	5790
chrX	17305750	17320386	*16A*	+	-	-	-	-	14636
chrX	17680734	17693452	*16D*	-	+	-	-	-	12718
chrX	17873436	17877060	*16F*	-	+	-	-	-	3624
chrX	18052522	18057452	*16F*	-	+	-	-	-	4930
chrX	18137912	18216512	*17A*	+	+	-	-	-	78600
chrX	18444238	18449382	*17C*	+	-	-	-	-	5144
chrX	18503812	18575040	*17C-17D*	-	+	-	-	-	71228
chrX	18815708	18890530	*17F-18A*	+	+	-	-	-	74822
chrX	19862460	20010890	*19A-19B*	+	+	-	-	-	148430
chrX	20185600	20230630	*19C*	-	+	-	-	-	45030
chrX	20470862	20868230	*19D-19E*	+	+	+	-	+	397368
chrX	21347022	21381730	*20A*	+	+	-	-	-	34708
chrX	21530644	21542948	*20A*	-	-	+	-	-	12304
chrX	21604638	21610666	*20B*	-	-	+	-	-	6028
chrX	21800385	21838058	*20C*	-	-	+	-	-	37673
chrX	21998874	22229684	*20C-20E*	+	+	+	-	-	230810
chr2L	654822	667994	*21E*	+	-	-	-	-	13172
chr2L	686046	700702	*21E*	-	+	-	-	-	14656
chr2L	1265972	1484372	*21F-22A*	+	+	-	-	-	218400
chr2L	3933064	4132202	*24D*	+	+	+	-	-	199138
chr2L	4485340	4756804	*24F-25A*	+	+	+	+	+	271464
chr2L	5205935	5212643	*25C*	-	-	+	-	-	6708
chr2L	5404364	5410432	*25E*	+	-	-	-	-	6068
chr2L	5439576	5446356	*25E*	-	-	+	-	-	6780
chr2L	6227234	6293084	*26B-26C*	+	-	+	-	-	65850
chr2L	8792023	8876820	*29F*	+	+	-	-	+	84797
chr2L	10592415	10641070	*32A*	-	-	+	-	-	48655
chr2L	11319650	11472460	*32E-32F*	+	+	-	-	+	152810
chr2L	11548122	11759130	*32F-33A*	+	+	+	-	-	211008
chr2L	12263834	12289382	*33D*	-	+	-	-	-	25548
chr2L	12795300	12928274	*34A*	-	+	+	-	+	132974
chr2L	14719634	14965740	*35B*	+	+	+	+	+	246106
chr2L	15306202	15653096	*35D*	+	+	+	-	+	346894
chr2L	15780110	16218532	*35D-35F*	+	+	+	+	+	438422
chr2L	16936380	17335190	*36B-36C*	+	+	+	+	+	398810
chr2L	17519484	18077158	*36C-36E*	+	+	+	+	+	557674
chr2L	18187610	18376652	*36E*	+	-	+	-	-	189042
chr2L	19250472	19304390	*37D*	-	+	-	-	-	53918
chr2L	20106646	20148632	*38C*	+	+	+	-	-	41986
chr2L	20223046	20232764	*38C*	-	-	+	-	-	9718
chr2L	20240270	20245622	*38C*	+	-	-	-	-	5352
chr2L	20526430	20576114	*38C*	+	+	-	-	-	49684
chr2L	21384238	21405566	*39D*	-	-	+	-	-	21328
chr2L	21556662	21560248	*39E*	-	-	+	-	-	3586
chr2L	21820966	22086308	*40A-40E*	-	+	+	-	+	265342
chr2L	22220028	22376472	*40F*	+	+	+	+	+	156444
chr2L	22578160	22617774	*40F*	-	+	-	-	+	39614
chr2L	22707402	22717322	*40F*	-	-	-	+	+	9920
chr2R	736214	956318	*41C-41D*	+	+	+	-	+	220104
chr2R	1046344	1055914	*41E*	-	-	+	-	-	9570
chr2R	1344406	1403847	*41F*	-	+	+	-	-	59441
chr2R	2186543	2215462	*42A*	-	-	+	-	-	28919
chr2R	2286252	2376122	*42A*	-	-	+	-	-	89870

chr2R	2411422	2444368	*42B*	-	+	-	-	-	32946
chr2R	7383072	7388597	*47F*	+	-	-	-	-	5525
chr2R	8743128	8753014	*49D*	-	-	+	-	-	9886
chr2R	9558256	9599556	*50C*	-	-	+	-	-	41300
chr2R	11626797	11628812	*52C*	+	-	-	-	-	2015
chr2R	12286556	12403585	*53C*	-	+	-	-	-	117029
chr2R	15742222	15844776	*56E*	-	+	+	-	-	102554
chr2R	15929940	16074850	*56F*	-	+	-	-	-	144910
chr2R	16322610	16393842	*57A*	-	+	-	-	-	71232
chr2R	17681724	17801086	*58A*	-	-	+	-	-	119362
chr2R	19020812	19024387	*59C*	-	-	-	-	+	3575
chr2R	19033882	19205342	*59D*	+	+	+	-	+	171460
chr2R	21046920	21143472	*60F*	-	-	+	-	-	96552
chr3L	20130	54020	*61A*	-	+	-	+	+	33890
chr3L	4719918	4722634	*64B*	-	-	+	-	-	2716
chr3L	4858616	5079226	*64C*	+	+	+	-	+	220610
chr3L	5396312	5476560	*64D*	-	-	+	-	-	80248
chr3L	6294762	6446982	*65A-65B*	+	+	+	-	+	152220
chr3L	6816574	6866124	*65D*	+	+	-	-	-	49550
chr3L	8484052	8488756	*66D*	+	-	-	-	-	4704
chr3L	9197216	9258644	*67A*	-	-	+	-	-	61428
chr3L	10014637	10149556	*67D*	+	-	+	-	-	134919
chr3L	10296522	10304055	*67D*	-	+	-	-	-	7533
chr3L	10481486	10602510	*67E*	-	+	-	-	-	121024
chr3L	10796044	10798652	*67F*	+	-	-	-	-	2608
chr3L	13089700	13193203	*70A*	-	+	+	-	-	103503
chr3L	13549876	13842484	*70B-70C*	+	+	+	+	+	292608
chr3L	14903194	14916010	*71A*	+	-	-	-	-	12816
chr3L	15200690	15450704	*71B-71D*	+	+	+	-	-	250014
chr3L	17173812	17191735	*73F*	-	-	+	-	-	17923
chr3L	18191100	18507442	*75C-75D*	+	+	+	-	+	316342
chr3L	20588140	20679702	*77D-77E*	+	+	+	-	-	91562
chr3L	22152882	22207742	*79C-79D*	-	+	-	-	-	54860
chr3L	22343290	22347274	*79E*	+	-	-	-	-	3984
chr3L	22491612	22606770	*79E-79F*	-	-	+	-	-	115158
chr3L	23377370	23611780	*80F*	+	+	-	+	+	234410
chr3L	23779140	23979920	*80F*	+	+	-	+	+	200780
chr3L	24063580	24374280	*>80F*	+	+	-	+	+	310700
chr3R	1919068	2131438	*83D-83E*	-	-	+	-	-	212370
chr3R	2333014	2427749	*83F*	+	-	+	-	-	94735
chr3R	2690295	2814586	*84A-84B*	-	+	+	-	+	124291
chr3R	3395104	3554983	*84D*	+	-	+	-	-	159879
chr3R	5550916	5560673	*85E*	-	-	+	-	-	9757
chr3R	6319864	6428203	*86C*	-	-	+	-	-	108339
chr3R	6439050	6455164	*86C*	+	-	-	-	-	16114
chr3R	6757546	6959123	*86C-86D*	+	+	+	-	-	201577
chr3R	7091662	7094668	*86D*	+	-	-	-	-	3006
chr3R	7921894	7926400	*87A*	-	-	+	-	-	4506
chr3R	7948122	7953310	*87A*	-	+	-	-	-	5188
chr3R	8643204	8712842	*87D*	+	-	-	-	-	69638
chr3R	9313440	9360472	*87F*	-	+	-	-	-	47032
chr3R	11418012	11493404	*88F-89A*	+	+	-	-	-	75392
chr3R	12505312	12774100	*89D-89E*	+	+	+	-	+	268788
chr3R	13054102	13143084	*89F-90A*	+	+	+	-	-	88982

chr3R	13712422	13718926	*90C*	+	-	-	-	-	6504
chr3R	13783982	13792660	*90D*	+	-	-	-	-	8678
chr3R	15982400	16005786	*92D*	-	-	+	-	-	23386
chr3R	16181286	16284194	*92E*	-	-	+	-	-	102908
chr3R	17947868	18120980	*94A*	+	+	+	-	-	173112
chr3R	18670914	18738876	*94D*	-	+	-	-	-	67962
chr3R	22131594	22200252	*97A-97B*	-	+	-	-	-	68658
chr3R	23188282	23296822	*97F-98A*	+	+	-	-	-	108540
chr3R	23851136	24100682	*98B-98C*	+	+	+	-	+	249546
chr3R	24250392	24278956	*98D*	+	+	-	-	-	28564
chr3R	26456226	26460270	*100A*	+	-	-	-	-	4044
chr3R	26467242	26569674	*100A*	-	+	+	-	+	102432
chr3R	26738740	26753934	*100A*	+	-	-	-	-	15194
chr3R	26795086	26801234	*100B*	-	-	+	-	-	6148
chr4	13824	25328	*N/A*	-	-	+	-	-	11504
			Total # URs:	89	103	71	12	31	
